# Metallothionein-3 Increases Triple-Negative Breast Cancer Cell Invasiveness via Induction of Metalloproteinase Expression

**DOI:** 10.1371/journal.pone.0124865

**Published:** 2015-05-01

**Authors:** Alicja M. Kmiecik, Bartosz Pula, Jaroslaw Suchanski, Mateusz Olbromski, Agnieszka Gomulkiewicz, Tomasz Owczarek, Anna Kruczak, Aleksandra Ambicka, Janusz Rys, Maciej Ugorski, Marzena Podhorska-Okolow, Piotr Dziegiel

**Affiliations:** 1 Laboratory of Glycobiology and Cell Interactions, Ludwik Hirszfeld Institute of Immunology and Experimental Therapy, Polish Academy of Sciences, Wroclaw, Poland; 2 Department of Histology and Embryology, Medical University, Wroclaw, Poland; 3 Department of Biochemistry, Pharmacology and Toxicology, Faculty of Veterinary Medicine, University of Environmental and Life Sciences, Wroclaw, Poland; 4 Department of Tumor Pathology, Maria Sklodowska–Curie Memorial Cancer Center and Institute of Oncology, Krakow, Poland; 5 Department of Physiotherapy, Wroclaw University School of Physical Education, Wroclaw, Poland; Columbia University, UNITED STATES

## Abstract

It has been recently found that metallothionein-3 (MT3) enhances the invasiveness and tumorigenesis of prostate cancer cells. This finding is in contrast to those of earlier studies, which indicated that overexpression of MT3 in breast cancer and prostate cancer cell lines inhibits their growth *in vitro*. Therefore, to clarify the role of MT3 in breast cancer progression, we analyzed the effect of MT3-overexpression on proliferation, invasiveness, migration, and tumorigenesis of breast cancer MDA-MB-231/BO2 cells. It was found that MDA-MB-231/BO2 cells overexpressing MT3 were characterized by increased invasiveness *in vitro*, compared to the control cells. Interestingly, this increased invasiveness correlated with a highly increased concentration of MMP3 in the culture supernatants (p<0.0001). Our data suggest that MT3 may regulate breast cancer cell invasiveness by modulating the expression of MMP3. These experimental results, obtained using triple-negative MDA-MB-231/BO2 cells, were further supported by clinical data. It was found that, in triple-negative breast cancer (TNBC), nuclear MT3 immunoreactivity in cancer cells tended to be associated with patients’ shorter disease-specific survival, suggesting that nuclear MT3 expression may be a potential marker of poor prognosis of triple-negative TNBC cases.

## Introduction

Metallothioneins (MTs) are a family of small (6–7 kDa), intracellular, non-enzymatic proteins that are highly conserved among species [1]. The polypeptide chain of MTs is characterized by high levels of cysteine, which enable the binding of metal ions such as Zn^+2^, Cd^+2^, Cu^+2^, and Hg^+2^ [2], and their subsequent transfer to the catalytic centers of various enzymes and binding sites for regulatory proteins [3,4]. Human MTs are encoded by a family of genes located on chromosome 16q13, and four main groups of these proteins, designated MT1–4, can be distinguished [5]. The ubiquitously expressed MT1 and MT2 have been shown to be involved in numerous important cellular processes in both normal and cancerous cells, such as the homeostasis of zinc ions, the detoxification of heavy metals, and the protection of cellular DNA against oxidative stress damage, proliferation, and apoptosis [1,2,4,6–8].

In contrast with MT1 and MT2, until recently MT3 was regarded as being tissue-specific, with expression restricted to neural tissues. It was first discovered in rat brain extracts in the course of Alzheimer’s disease [9], and then in normal astrocytes and neurons [10]. However, MT3 expression has also been reported in renal epithelial cells (including proximal tubules) [11,12] and in some epithelial cells of the normal human prostate [13]. In addition to normal tissues, high expression of MT3 has been reported in several types of cancers, including urinary bladder, breast, prostate and non-small cell lung cancers [13–16]. However, decreased expression of MT3 has been found in gastric cancer, esophageal adenocarcinoma, and squamous cell cancer [17–19].

It was originally reported that MT3 possesses a unique neuronal cell growth inhibitory property [9,20,21]. However, the inhibition of cell proliferation has also been observed in the case of some breast cancer cell lines. Gurel *et al*. found that overexpression of MT3 inhibited the growth of MCF-7 and Hs578T breast cancer cells, while not affecting the proliferation rates of two other cell lines, T47D and MDA-MB-231 [22]. Contradictory results have been obtained with prostate cancer cells. Stable transfection of PC-3 prostate cancer cells with MT3 resulted in their reduced growth rates [23]. On the other hand, it was recently shown that MT3 overexpression in the same PC-3 prostate cancer cells enhances cell growth *in vitro* and tumorigenesis *in vivo* [24]. In addition, these cells were characterized by increased migration and invasion *in vitro*.

Taken together, the role of MT3 in cancer progression remains ambiguous and inconsistent. Therefore, in the present study, the impact of MT3 overexpression on the proliferation, migration, *in vitro* invasiveness, and tumorigenesis of breast cancer MDA-MB-231/BO2 cells was studied. Furthermore, using the immunohistochemical (IHC) method, MT3 expression was studied in a series of triple-negative breast cancers (TNBC), which are devoid of estrogen (ER) and progesterone (PR) receptors, and human epidermal growth factor receptor-2 (HER-2) expression with regard to patients’ clinical and pathological data.

## Materials and Methods

### Cell lines

The human breast cancer cell lines: MCF-7, MDA-MB-231 (ATCC, Washington, CO, USA), its derivative MDA-MB-231/BO2 (courtesy of Dr. Philippe Clezardin, INSERM U664, France) [25], SK-BR-3, and BT-474 (from the Cell Line Collection of the Ludwik Hirszfeld Institute of Immunology and Experimental Therapy, Polish Academy of Science, Wroclaw, Poland) were cultured in α-minimum essential medium (α-MEM) supplemented with 10% fetal calf serum (FCS; Invitrogen, Carlsbad, CA, USA), 2 mM L-glutamine, 100 U/ml streptomycin, and 0.1 mg/ml penicillin (complete α-MEM). Human immortalized normal breast cells (hTERT-HME1; ATCC) were cultured in MEGM Bulletkit medium (Lonza, Basel, Switzerland).

### Triple-negative breast cancer (TNBC) samples

The use of clinical tumor samples was approved by the Commission of Bioethics at Wroclaw Medical University (Wroclaw, Poland). All the patients gave written informed consent for use of the samples in the experimental study. TNBC (51 cases) formalin-fixed paraffin embedded tumors were sampled at the Department of Tumor Pathology, Centre of Oncology, Maria Sklodowska–Curie Memorial Institute, Krakow, Poland. The clinical and pathological traits of the patients are presented in Table 1. The mean patients’ age at diagnosis was 51.59 ± 12.08 years (range 35–83). The patients were treated by mastectomy or quadrantectomy, with a subsequent axillary lymph node resection. In six cases (11.8%) neoadjuvant chemotherapy prior to surgical resection of the tumors was applied. Forty eight patients (94.1%) received adjuvant chemotherapy, whereas radiotherapy was administered to 33 (64.7%). The patients were followed up for 68.5 ± 49.1 months (range 1–196 months). During this period, ten of the patients (19.6%) died of the disease.

**Table 1:** Clinical and pathological characteristics of the 51 triple-negative breast cancer (TNBC) cases.

Parameter	No.	%
Age		
≤50	27	52.9
>50	24	47.1
Menopausal status		
Pre	26	51.0
Post	24	47.1
NA	1	1.9
Tumor size		
pT1	14	27.5
pT2	29	56.9
pT3	7	13.7
pT4	1	1.9
Lymph nodes		
pN-	26	51.0
pN+	25	49.0
pTNM		
I	8	15.7
II	16	58.8
III	11	21.6
IV	2	3.9
Malignancy grade		
G1	1	1.9
G2	13	25.6
G3	37	72.5
Necrosis		
Absent, low	29	56.9
Moderate, intense	22	44.1
Fibrosis		
Absent, low	13	25.5
Moderate, intense	38	74.5
Tumor infiltrating lymphocytes		
Absent, low	14	27.5
Moderate, intense	37	72.5

NA—not available

From the obtained TNBC samples, 6-μm-thick haematoxylin-eosin (H&E) sections were made and a tumor malignancy grade (G) was assessed by two pathologists, according to the modified criteria of Elston and Ellis [26]. Furthermore, in these sections the extent of tumor necrosis, fibrosis and tumor infiltrating lymphocytes were evaluated semi-quantitatively and encoded as follows: 0 (absent), 1 (weak), 2 (moderate) and 3 (intense) (Table 1).

### Construction of vectors, virus production, and transductions

To construct the MT3 expressing vector, first the IRES sequence derived from the pWP1 vector (kindly provided by Dr. D. Trono, École Polytechnique Fédérale de Lausanne, Switzerland) and *Photinus pyrolis* luciferase cDNA derived from pGL3 vector (Promega, Fitchburg, WI, USA) were cloned into pRRL-cPPT-CMV-X2-PRE-SIN vector (D. Trono, École Polytechnique Fédérale de Lausanne, Switzerland), in order to obtain a construct named pRRL-IRES-LUC. Then, a DNA cassette containing the puromycin N-acetyl-transferase (PAC) cDNA, 2A sequence, and MT3 cDNA, excised from the pCR2.1-PUR-2A-MT3 vector (GeneART, Life Technologies, Carlsbad, CA, USA), was cloned into the pRRL-IRES-LUC vector. The resulting construct was named pRRL-PURO-2A-MT3-IRES-LUC. The control pRRL-PURO-IRES-LUC vector was obtained by cloning puromycin N-acetyl-transferase (PAC) cDNA, excised from a pPUR vector (Clontech, Terra Bella Avenue Mountain View, CA, USA), into the pRRL-IRES-LUC vector.

For lentivirus production and packaging, HEK 293T cells were cotransfected at 40–60% confluence with 10 μg pRRL-PURO-2A-MT3-IRES-LUC or 10 μg pRRL-PURO-2A IRES-LUC, 5 μg pMDL-g/p-RRE, 2.5 μg pRSV-REV, 3 μg pMk-VSVG (D. Trono, École Polytechnique Fédérale de Lausanne, Switzerland), using polyethylenimine (Sigma-Aldrich, Buchs, Switzerland) at a concentration of 1 mg/mL. The virus-containing supernatant was concentrated 100× on an Amicon Ultra-15K:100.000 (Millipore, Billerica, MA, USA).

The MDA-MB-231/BO2 cells (2×10^4^) were transduced with the concentrated virus stock by centrifuging (2460×g) at 23°C for 2 hours. Following overnight incubation, the medium was replaced with fresh complete α-MEM.

### SiRNA transfections

Transfections with 4 different MMP3 siRNA (NM_002422.3) or 4 different MT3 siRNA (NM_005954.2) were performed according to Fast-Forward Protocol Reverse-Transfection Protocol (Qiagen, Hilden, Germany). Briefly, cells (2.5×10^5^/well) seeded, 30 minutes before transfection, in 6-well plates (Greiner Cellstar, Sigma-Aldrich) in complete α-MEM, were incubated with specific siRNA or nonspecific control siRNA. The transfection reagent was prepared by diluting 150 ng/6 μl of appropriate siRNA and 12 μl of HiPerfect Reagent (Qiagen) with 82 μl of α-MEM. After 48 h incubation, cells were harvested by trypsinization and subjected to further experiments.

### Reverse transcription polymerase chain reaction (RT-PCR) and quantitative real-time PCR (qPCR)

The total RNA from the cell lines under study was extracted using an RNeasy Mini Kit (Qiagen), according to the manufacturer’s protocol. The reverse transcriptase reaction was performed using a SuperScript III First Strand synthesis System (Invitrogen). Briefly, 1 μg of total RNA was mixed with 2.5 μM oligo(dT), 0.5 mM dNTPs, and DEPC-treated water. The sample was incubated at 65°C for 5 min, and placed on ice for 1 min. The reaction mixtures, containing reverse transcription buffer, 5 mM MgCl_2_, 10 mM DTT, 40 U RNase inhibitor, and 200 U SuperScript III reverse transcriptase, were incubated at 50°C for 50 min, terminated at 85°C for 5 min, and chilled on ice. At the end, 2 U of RNase H were added and incubated at 37°C for 20 min.

The expression of MT3 in the studied cell lines was determined by RT-PCR using a Taq PCR Core Kit (Qiagen). The reaction mixture contained 1×PCR Buffer, 1.5 mM MgCl_2_, 200 μM dNTPs, 0.2 μM of each primer, and 1.25 U Taq DNA Polymerase. The specific primers for MT3 mRNA (F: 5’-CTATGGACCCAGAGACA-3’, R: 5’-TTGGCACACTTTTCACACT-3’) and β-actin mRNA (F: 5’ ACCACACCTTCTACAATGAGC 3’, R: 5’ GATAGCACAGCCTGGATAGC 3’) were obtained from the Institute of Biochemistry and Biophysics, Polish Academy of Sciences, Warsaw. The reaction conditions were as follows: initial denaturation at 94°C for 3 min, 35 cycles of denaturation at 94°C for 30 s, annealing at 58°C for 30 s, and elongation at 72°C for 30 s, followed by final extension at 72°C for 10 min. The PCR products were subjected to electrophoresis in 2% agarose gel containing ethidium bromide and visualized in a ChemiDoc XRS System (Biorad, Hercules, CA, USA).

The mRNA expression of MT3, MMP1, MMP2, MMP3, MMP9, and TIMP1 was determined by qPCR with 7500 Real-Time PCR System and TaqMan Gene Expression Master Mix (Applied Biosystems, Waltham, MA, USA), according to the manufacturer’s protocols. β-actin was used as a reference gene. The following primers and TaqMan probes were used: MT3 Hs00359394_g1, MMP1 Hs00899658_m1, MMP2 Hs01548727_m1, MMP3 Hs00968305_m1, MMP9 Hs00234579_m1, TIMP1 Hs00171558_m1 and β-actin Hs99999903_m1 (Applied Biosystems). All reactions were performed in triplicate under the following conditions: activation of polymerase at 50°C for 2 min, initial denaturation at 94°C for 10 min, followed by 40 cycles of denaturation at 94°C for 15 s, and annealing with elongation at 60°C for 1 min. The relative expression of the studied genes was calculated with the ∆∆Ct method.

### SDS-PAGE and Western blot

Whole cell lysates were obtained using a CelLytic solution (Sigma, St. Louis, MI, USA) with the addition of a Protease Inhibitor Cocktail (Sigma) and 0.2 mM phenylmethanesulfonylfluoride (PMSF). In order to obtain the nuclear and cytoplasmic fractions, the cells were treated with ProteoExtract Subcellular Proteome Kit (Merck, Darmstad, Germany) according to the manufacturer’s instructions. The proteins were quantified using a BCA assay kit (Pierce, Rockford, IL, USA). The lysates of the whole cells, as well as the nuclear and cytoplasmic fractions (corresponding to 30 μg of protein), were mixed with the sample buffer containing dithiothreitol (DTT), and resolved using SDS-PAGE. Following the completion of electrophoresis, the samples were transferred to polyvinylidene fluoride (PVDF) membranes (Immobilon, Millipore), which were treated with 4% BSA solution in TBS containing 0.1% Tween-20 (Invitrogen). Next, for the detection of MT3, the membranes were incubated with anti-human MT3 rabbit polyclonal antibodies raised against the GGEAAEAEAEKC peptide (Invitrogen) (1:1000). Rabbit polyclonal antibodies were used for detection of MMP1 (1:400; ProteinTech, Chicago, IL, USA), MMP3 (1:800; Abgent, San Diego, CA, USA), MMP9 (1:400; Dako, Glostrup, Denmark), and calpain (1:500; ProteinTech). Murine monoclonal antibody was used to detect the presence of MMP2 (1:800; Abgent). Primary antibodies were incubated overnight at 4°C, and then with horseradish peroxidase (HRP)-conjugated donkey polyclonal antibodies directed against rabbit and murine immunoglobulins (Jacksons Immunoresearch, West Grove, PA, USA) at room temperature (RT) for 1 hour. After rinsing with TBS buffer containing 0.1% Tween-20, the blots were incubated with the Immun-Star-HRP Chemiluminescent Substrate (Biorad). The quantifications of cytoplasmic proteins were based on β-actin expression determined by utilizing anti-human β-actin murine monoclonal antibody (Cell Signaling, Danvers, MA, USA). The quantifications of nuclear proteins were based on histone H3 expression, determined by utilizing rabbit polyclonal anti-histone H3 antibodies (Cell Signaling, Beverly, MA, USA). The purity of nuclear and cytoplasmic fractions was demonstrated by the lack of binding of anti-calpain antibodies to nuclear proteins and anti-histone H3 antibodies to cytoplasmic proteins (**S1 Fig**).

### MTT assay

For the MTT assay, cells (3×10^3^/well) were seeded on a 96-well plate and grown in complete α-MEM for 5 days. Every 24 hours, 50 μl of thiazolyl blue tetrazolium bromide solution (MTT; 5 mg/ml) (Sigma) was added to each well, and the cells were cultured at 37°C, 5% CO_2_ for 2 hours. After this time, the medium was removed and 0.1 ml of DMSO was added to dissolve MTT-formazan; after an additional 2 hours, the absorbance was measured at a wavelength of 550 nm. The reference wavelength was 630 nm (EL311 Microplate Reader, Behring, King of Prussia, PE, USA). The experiments were performed in triplicates and repeated three times.

### Migration and invasion assay

The migration and invasion assays were performed using, respectively, a BD Falcon FluoroBlok 24 Multiwell Insert System and a BD BioCoat Tumor Invasion System (Becton Dickinson, Franklin Lakes, NJ, USA). Cells were labeled with 2.5 μM Cell Tracker (Invitrogen) at 37°C for 30 min. For the invasion assay, the plate was removed from -20°C storage and allowed to come to RT. 500 μL of warm (37°C) PBS was added to the interior of the insert wells and left for 2 hours at 37°C, 5% CO_2_, to rehydrate. After rehydration, PBS was removed from the insert well. The cells were collected by trypsinization and resuspended in α-MEM at a density of 5×10^4^ cells/mL. Then 500 μL of cell suspension (2.5×10^4^) was added to the apical chambers, while 750 μL of chemoattractant (5% FBS in α-MEM) was added to each of the basal chambers. The cells were incubated at 37°C, 5% CO_2_, and the fluorescence was measured every 5 hours. The fluorescence of the cells was read at wavelengths of 485 and 538 nm (for excitation and emission, respectively) on a bottom-reading fluorescent microplate reader (Fluoroscan Ascent FL, Thermo Scientific, Waltham, MA, USA). The migration assay was performed utilizing the same protocol, but without the rehydration step. These experiments were independently performed three times.

### ELISA assay

The Quantikine Human Total MMPs Immunoassay (R&D Systems, Minneapolis, MN USA) was used to detect MMP1, MMP2, MMP3, MMP9, and TIMP1 in the cell culture supernatants. The assay was performed according to the manufacturer’s instructions.

### Immunofluorescence imaging

Cells were fixed with 4% paraformaldehyde for 12 min at RT, and permeabilized using 0.2% Triton-X for 10 min. The cells were then incubated for 18 hours at 4°C with primary anti-MT3 rabbit polyclonal antibodies (Invitrogen), diluted 1:800. Subsequently, fluorescein isothiocyanate (FITC)-conjugated goat F(ab’)_2_ fragment against rabbit immunoglobulins was applied (1:50, 1 h, RT) (Jacksons Immunoresearch). The sections were mounted in a DAPI-containing medium (Vectashield Mounting Medium; Vector Laboratories, Burlingame, CA, USA) and analyzed using a BX51 fluorescence microscope and Cell^F^ software (Olympus, Tokyo, Japan). The respective negative controls were performed by omitting the addition of primary antibody.

### Animals and tumor growth

Female athymic Crl:NU-Foxn1^nu^ mice, 8–10 weeks old, were obtained from Charles River (Wilmington, MA, USA) and kept under pathogen-free conditions. Animal experiments were performed according to the International Animal Care Convention, and all experimental protocols described in this study were approved by the First Local Ethic Committee for Animal Experimentation (No. 55/2009; Wroclaw, Poland). Human breast cancer cells were harvested using 0.05% trypsin/0.02% EDTA, washed with PBS and resuspended in the same buffer. Cell suspensions (2×10^6^/100 μl PBS) were mixed with the same volume of ice-cold BD Matrigel Matrix High Concentration (Becton Dickinson), and the whole mixture was inoculated subcutaneously (*s*.*c*.) proximal to the mammary fat pad. During this procedure, and all others (see below), the mice were anesthetized with a mixture of isoflurane and oxygen.

Tumor growth was monitored using caliper measurements, and tumor volume was calculated using the formula (a^2^×b)/2, where *a* is the shorter diameter in mm and *b* is the longer diameter in mm.

Observations were carried out once a week between the fourth and the tenth weeks following cell injection. The body condition score (BCS) technique was used to assess the health status of the experimental mice as part of a daily routine. Mice with cachexia symptoms (BCS = 1) were sacrificed by cervical dislocation after light anesthesia with isofluorane inhalation. The tumor tissues collected in 10% buffered formalin were subjected to histological studies and processed routinely. Six-μm-thick sections were stained with H&E, and blindly examined by two independent pathologists.

### Immunhistochemistry (IHC)

Immunohistochemical reactions were performed, as described elsewhere [16], on 4-μm-thick paraffin sections fixed on microscopic slides (SuperFrost+; Menzel-Glässer, Braunschweig, Germany). Deparaffinization and antigen retrieval were then performed using Antigen Retrieval Solution (97°C, 20 min; Dako) in a PT Link Rinse Station (Dako). Following this, the sections were pretreated with a FLEX Target Retrieval Solution pH 9.0 for all the studied antigens, with the exception of the Ki-67 antigen, for which the pH 6.0 buffer was used. Subsequently, the sections were washed in TBS/0.05% Tween and incubated with EnVision FLEX Peroxidase-Blocking Reagent (5 min at RT), followed by the next washing step with TBS/0.05% Tween. Then the primary antibodies were applied for 20 min at RT in a Dako Autostainer Link48, in order to ensure stable and repeatable reaction conditions. MT3 expression was studied using rabbit polyclonal antibodies raised against GGEAAEAEAEKC peptide (Invitrogen, dilution 1:800), whereas Dako prediluted murine monoclonal antibodies were utilized to assess the expression of Ki-67 antigen (MIB-1), estrogen receptor (ER, clone 1D5), and progesterone receptor (PR, clone 636). The sections were than washed in TBS/0.05% Tween, and EnVision FLEX/HRP secondary antibodies (Dako) were applied for 20 min at RT. The peroxidase substrate, diaminobenzidine (DAB), was applied and the sections were incubated for 10 min at RT in order to visualize the studied antigens. Finally, the sections were counterstained with Mayer’s haematoxylin, dehydrated in alcohol (70%, 96%, 99.8%) and xylene, and mounted in the SUB-X Mounting Medium (Dako).

Human epidermal growth factor receptor-2 (HER2) expression was assessed using the HercepTest kit (Dako), according to the procedure recommended by the manufacturer. In cases of equivocal IHC results (+2), an HER2 FISH pharmDx Kit (Dako) was utilized to determine the HER2 amplification status. All slides were counterstained with haematoxylin (Dako), and all the reactions were conducted with negative controls in which the primary antibodies were omitted.

### Terminal Deoxynucleotidyl Transferase dUTP Nick End Labeling (TUNEL)

Apoptosis detection was performed using an ApopTag Peroxidase In Situ Apoptosis Detection Kit (Millipore). Briefly, paraffin sections were deparaffinized in xylene, rehydrated in alcohol, and rinsed in distilled water and PBS, pH 7.4. Then the sections were incubated with Proteinase K (Dako) for 5 min at RT and rinsed in PBS. The activity of endogenous peroxidase was blocked by 5 min incubation in 3% H_2_O_2_/PBS. In the next step, the sections were incubated in Equilibration Buffer for 10 min at RT, with a subsequent incubation with TdT Enzyme and Reaction Buffer at 37°C for 1 h. The reaction was blocked after 10 min of incubation in the Stop Buffer and rinsed in PBS, followed by the application of antidioxygenin peroxidase-conjugated antibody for 30 min at RT. Next, the sections were incubated for 10 min with DAB to visualize the TUNEL-positive cell nuclei. Finally, the sections were counterstained with Mayer’s hematoxylin, dehydrated in alcohol, and mounted in SUB-X Mounting Medium (Dako).

### Evaluation of IHC reactions and TUNEL

IHC reactions of the experimental tumors and human TNBC cases were assessed under a BX41 light microscope (Olympus) by two independent pathologists blinded to the experimental and patient data. The expression of MT3 in cancer cell nuclei was assessed in whole TNBC tissue sections, as described elsewhere [16], and encoded as follows: 0 (0% cells stained), 1 (1%–10% cells stained), 2 (11%–25% cells stained), 3 (26%–50% cells stained), 4 (51%–100% cells stained). The Ki-67 antigen and TUNEL sections from the induced tumors were assessed in three hotspots (areas of potentially highest expression of the examined marker) under ×400 magnification with a computer image analysis system, Cell^D^ (Olympus) [27]. For each examined hotspot, the ratio of positively stained tumors cells to all tumor cells was estimated, and the average mean percentage of the three hotspots was used for the purpose of the statistical analysis.

### Statistical analysis

Prism 5.0 software (GraphPad, La Jolla, CA, USA) was utilized to analyze the data. The Mann–Whitney test was used to compare the groups of data that did not meet the assumptions of the parametric test. Two-way ANOVA with the Bonferroni multiple comparison test was used to analyze the differences between the analyzed cell lines. The Kaplan-Meier method and the log-rank test were employed to determine the significance of the clinical and pathological traits on disease-specific survival. For each variable, the hazard ratio and the 95% confidence interval (95% CI) were estimated. In all analyzes, the results were considered statistically significant when p<0.05.

## Results

### Generation of MT3-overexpressing breast cancer cell line

**Fig 1:**
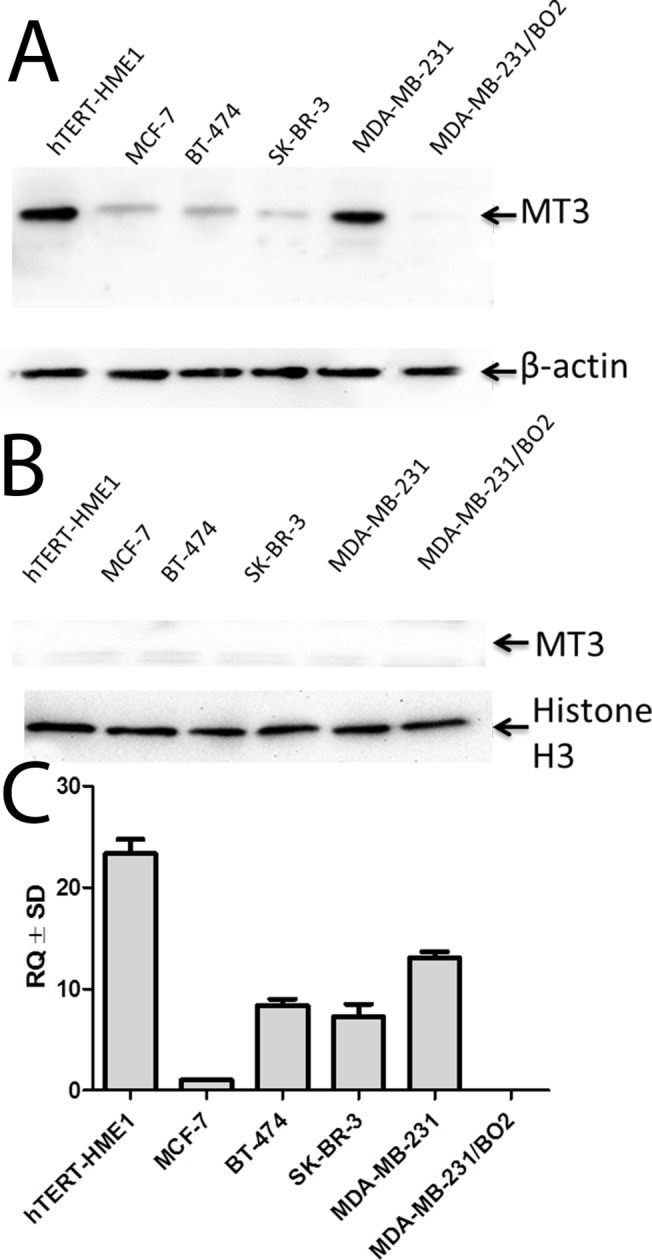
Characteristics of breast cancer cell lines according to MT3 expression. Western blot analysis using anti-MT3 rabbit polyclonal antibodies binding to **(A)** cytoplasmic and **(B)** nuclear proteins of breast cancer cell lines. Cell lysates equivalent to 30 μg of protein were separated by SDS-PAGE under reducing conditions on a 12% gel and electrophoretically transferred onto a nitrocellulose membrane. β-Actin and histone H3 served as an internal controls, respectively, for cytoplasmic and nuclear proteins. Expression of MT3 mRNA in breast cancer cell lines **(C)**. Real-time PCR was used to analyze MT3 mRNA. Relative expression (RQ) of *MT3* gene was normalized against expression of *ACTB* gene and cell line MCF7 was assigned as a calibrator sample. Results are expressed as mean ± SD.

**Fig 2:**
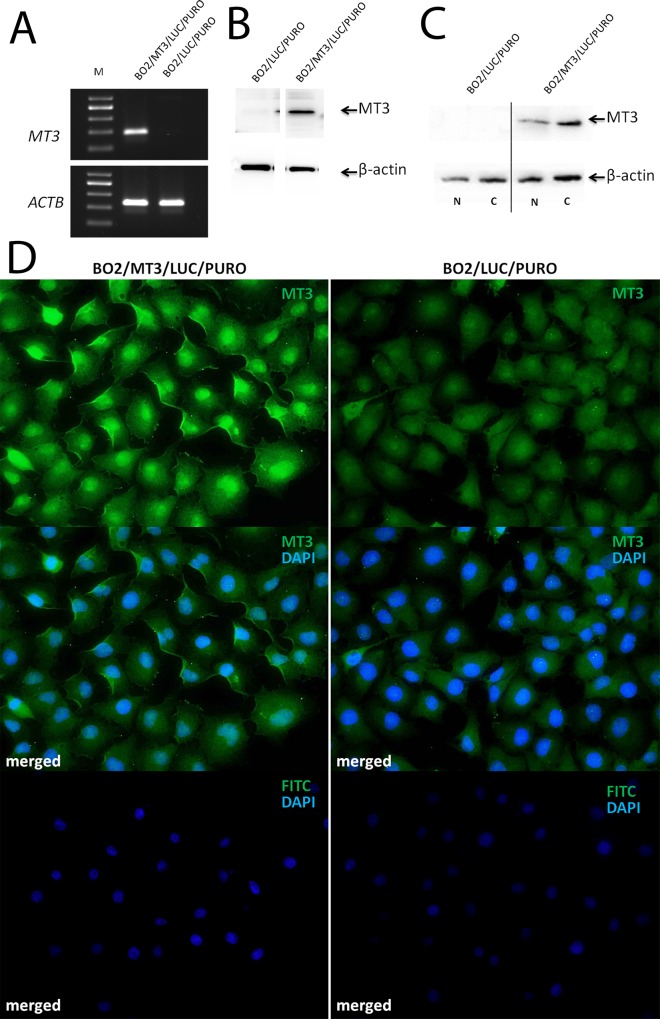
Characteristic of BO2/MT3/LUC/PURO cells overexpressing MT3. **(A)** RT-PCR analysis of MT3 mRNA from control BO2/LUC/PURO cells and BO2/MT3/LUC/PURO cells overexpressing MT3. *ACTB* was used as reference gene. M—DNA marker. **(B)** Western blot analysis using anti-MT3 rabbit polyclonal antibodies binding to whole cell lysates. **(C)** Western blot analysis using anti-MT3 rabbit polyclonal antibodies binding to cytoplasmic (*C*) and nuclear proteins (*N*) of control BO2/LUC/PURO and MT3-overexpressing BO2/MT3/LUC/PURO cells. Cell lysates equivalent to 30 μg of protein were separated by SDS-PAGE under reducing conditions on a 12% gel and electrophoretically transferred onto a nitrocellulose membrane. **(D)** Immunofluorescent imaging. Binding of anti-MT3 rabbit polyclonal antibodies to BO2/MT3/LUC/PURO cells overexpressing MT3 and control BO2/LUC/PURO cells. Negative controls were performed by omitting the primary antibody. Secondary FITC-conjugated rabbit antibody was applied to visualize the antigens. Magnification ×400.

In order to create a gain-of-function cellular model based on the stable overexpression of MT3, we screened the cultured noncancerous (hTERT-HME1) and breast cancer cell lines (MCF-7, SK-BR-3, BT-474, MDA-MB-231 and MDA-MB-231/BO2) for the expression of MT3 mRNA and protein. As previous reports have revealed both cytoplasmic and nuclear localization of MT3 [16,24], both fractions were analyzed separately by Western blot with rabbit polyclonal antibodies directed against MT3. High levels of MT3 were found in the cytoplasmic fractions of hTERT-HME1 and MDA-MB-231 cells, and low amounts of the protein were present in the cytoplasmic fractions of MCF-7, SK-BR-3 and BT-474 cells. There was no expression of MT3 in MDA-MB-231/BO2 cells (**Fig 1A**). Only residual amounts of MT3 were found in their nuclear fractions (**Fig 1B**). Moreover, no MT3 mRNA was detected in MDA-MB-231/BO2 cells (**Fig 1C**). As MDA-MB-231/BO2 cells did not produce MT3, they were chosen to construct a gain-of-function phenotype. To do so, the cells were transduced with the pRRL-PURO-2A-MT3-IRES-LUC expression vector, and resulting cell population, which overexpressed MT3 on the mRNA and protein levels, was named BO2/MT3/LUC/PURO (**Fig 2A and 2B).** In the transduced cells, MT3 was found not only in the cytoplasm, but also in nucleus, as shown by Western blot (**Fig 2C**) and immunofluorescence imaging (**Fig 2D**). Control BO2/LUC/PURO cells with no expression of MT3 were obtained by transduction of MDA-MB-231/BO2 cells with the pRRL-PURO-2A IRES-LUC vector.

### Effect of MT3 expression on breast cancer cells growth in vitro and tumorigenesis

**Fig 3:**
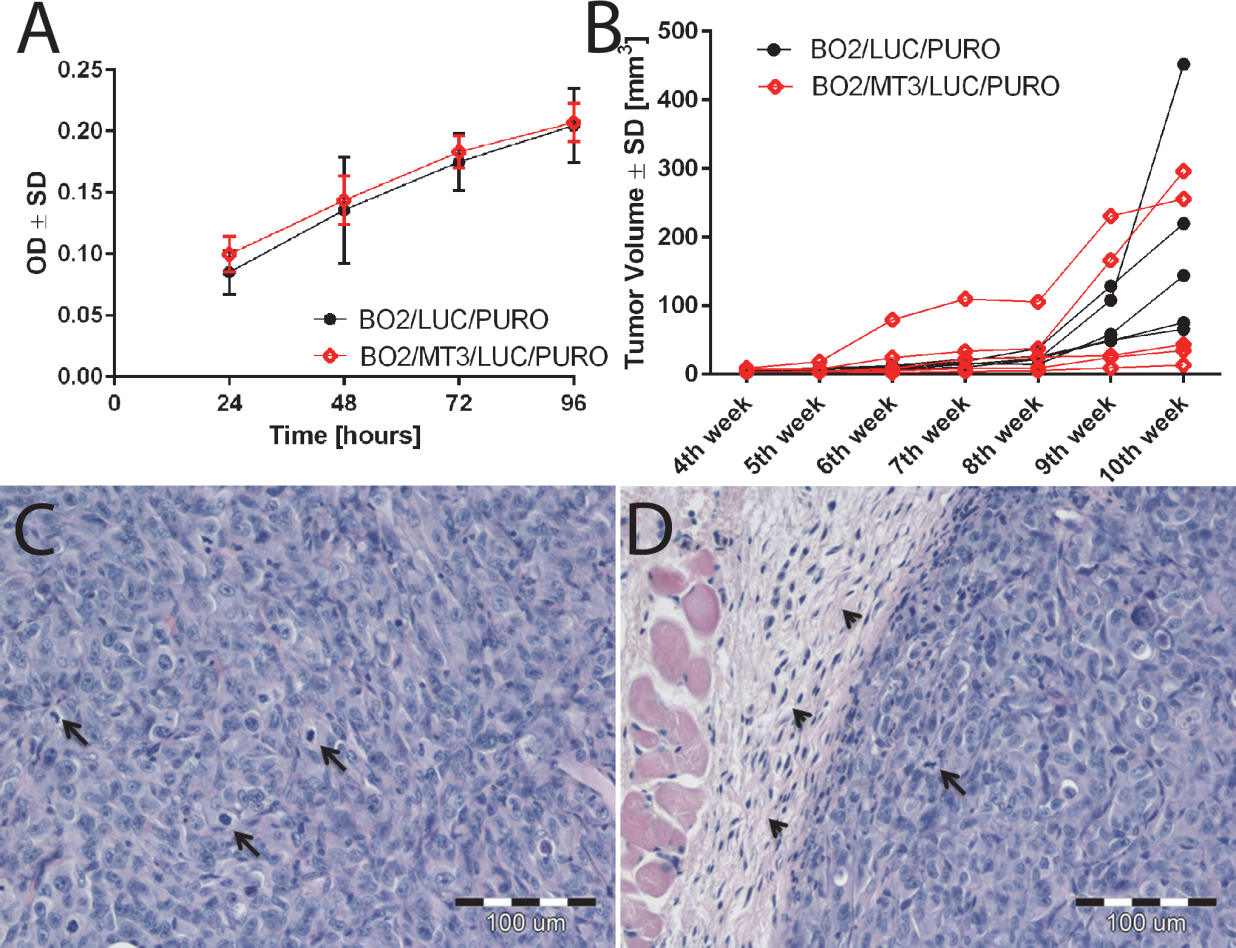
Effect of MT3 expression on breast cancer cells growth *in vitro* and tumorigenesis. **(A)** Proliferation of control BO2/LUC/PURO cells and BO2/MT3/LUC/PURO cells overexpressing MT3. Cell proliferation was determined *in vitro* using MTT assay, as described in the “Materials and Methods”. The values are shown as the mean ± SD of three independent experiments. **(B)** Xenograft tumor growth of control BO2/LUC/PURO cells and BO2/MT3/LUC/PURO cells overexpressing MT3. Tumor growth was recorded on a weekly basis using metric calipers. Data are shown as the mean tumor volume for each group of mice (n = 5) at each indicated time point. **(C)** Haematoxylin and eosin stained tumor sections presenting spindle-like cancer cells with visible mitotic figures and **(D)** fibrous capsule surrounding the tumor mass. Arrows indicate mitotic figures (**C** and **D**); arrowheads, fibrous capsule (**D**). Magnification ×200.

It was recently shown that MT3 increases proliferation and enhances tumorigenesis of prostate cancer cells [24]. Therefore, to determine whether overexpression of MT3 affects the proliferation potential of breast cancer cells, BO2/MT3/LUC/PURO and control BO2/LUC/PURO cells were subjected to MTT assay. It was found that the growth rate was essentially the same for both cell types (**Fig 3A**). To assess further whether the presence of MT3 might affect the tumorigenic properties of breast cancer cells, BO2/MT3/LUC/PURO and control BO2/LUC/PURO cells were transplanted subcutaneously into nude mice. At the end of the experiment (after 10 weeks), no significant differences were observed in the tumor volumes generated by both cell types (**Fig 3B**). Histological analysis of the H&E-sections revealed that both cell types formed tumors composed of proliferating spindle-like cells with focal areas of necrosis, surrounded by a fibrous capsule (**Fig 3C and 3D**). Analysis of the Ki-67 antigen expression and TUNEL cell positivity revealed no differences between the tumors generated by MT3-overexpressing BO2/MT3/LUC/PURO cells and the control BO2/LUC/PURO cells (**S2 Fig**).

### MT3 expression affects invasiveness of breast cancer cells in vitro

**Fig 4:**
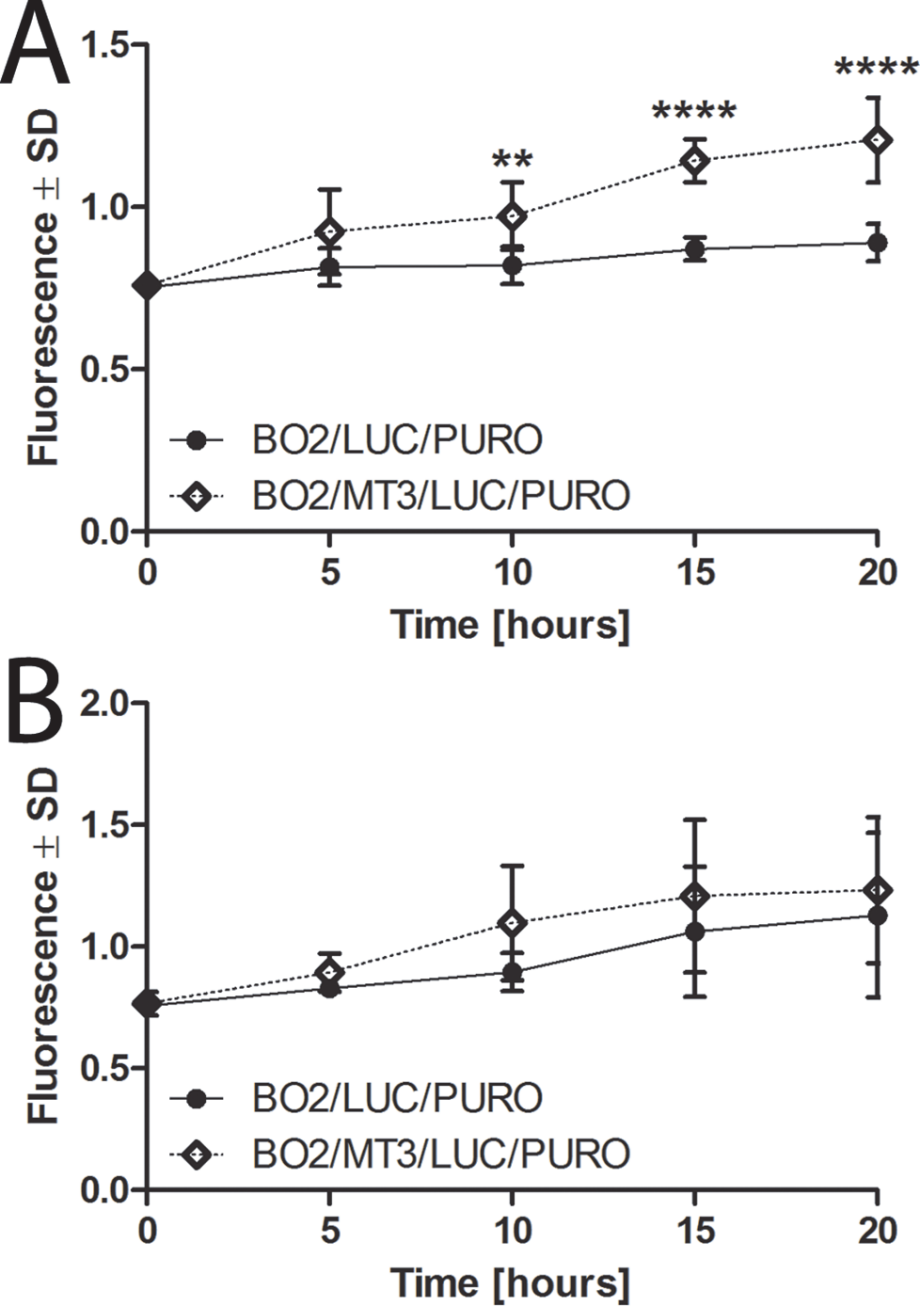
MT3 expression affects invasiveness of breast cancer cells *in vitro*. Invasiveness **(A)** and migratory capability **(B)** of BO2/MT3/LUC/PURO and BO2/LUC/PURO cells. Data are presented as mean ± SD. Bonferroni multiple comparison test, **p<0.01, ****p<0.0001.

Several lines of evidence suggest that metallothioneines affect the invasive properties of prostate and breast cancer cells [24,28]. Therefore, the BO2/MT3/LUC/PURO cells expressing MT3 and the control BO2/LUC/PURO cells were subjected to *in vitro* matrigel invasion assay. A statistically significant increase in invasiveness was found in the case of breast cancer cells overexpressing MT3 in comparison to the control cells at the 10^th^, 15^th^, and 20^th^ hours of the experiment (p<0.01, p<0.0001 and p<0.0001, respectively; Bonferroni multiple comparison test) (**Fig 4A**). However, when the migration properties of both cell types were compared, no differences were observed in *in vitro* migration assay (**Fig 4B**).

### Expression of MMPs and TIMP1 in analyzed cell lines

**Fig 5:**
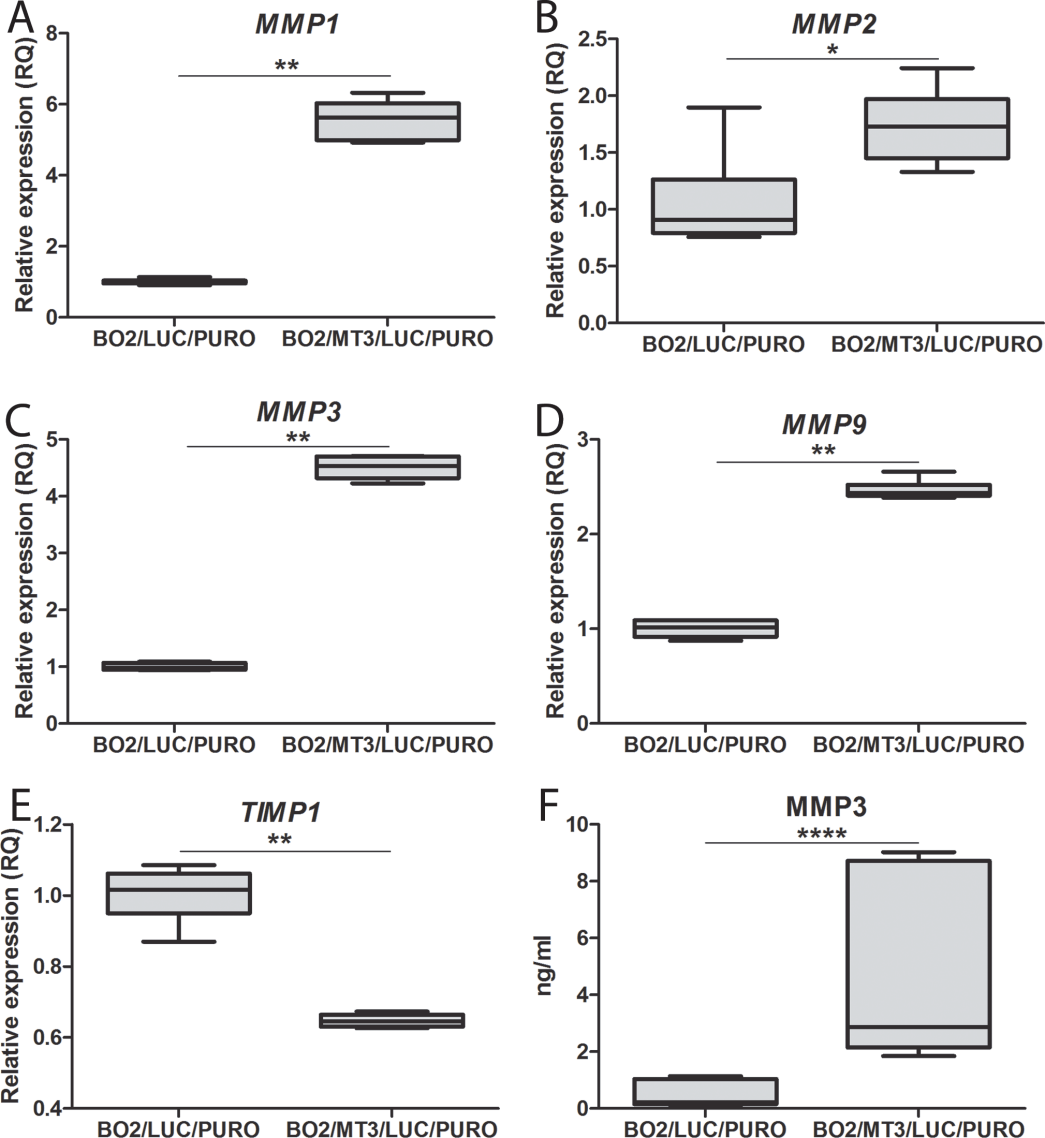
Expression of MMPs and TIMP1 in control BO2/LUC/PURO cells and BO2/MT3/LUC/PURO cells overexpressing MT3. **(A)** MMP1, **(B)** MMP2, **(C)** MMP3, **(D)** MMP9 and **(E)** TIMP1 mRNAs Real-time PCR was used to analyze MT3 mRNA. Relative expression (RQ) of *MT3* gene was normalized against expression of *ACTB* gene and BO2/LUC/PURO cells were assigned as a calibrator sample. Results are expressed as mean ± SD. **(F)** MMP3 protein level in culture media of control BO2/LUC/PURO cells and BO2/MT3/LUC/PURO cells overexpressing MT3. The Quantikine Human Total MMPs Immunoassay (R&D Systems, Minneapolis, MN USA) was used to detect MMP3. Mann–Whitney test, *p<0.05, **p<0.01, ***p<0.001.

In the breast cancer MDA-MB-231 cells, their enhanced invasive properties, caused by the overexpression of the MT2A isoform, were associated with the increased expression of MMP-9 [28]. Based on this result, we analyzed the expression of several metalloproteinases in MDA-MB-231/BO2 cells that overexpress MT3 and in control MDA-MB-231/BO2 cells transduced with an empty vector. Quantitative PCR analysis revealed a significant increase in *MMP1* (p<0.01), *MMP2* (p<0.05), *MMP3* (p<0.01), and *MMP9* (p<0.01) gene expression when the BO2/MT3/LUC/PURO cells overexpressing MT3 were compared with the BO2/LUC/PURO cells (Mann–Whitney test) (**Fig 5A–5D**). Therefore, the expression of metalloproteinases was further analyzed in the supernatants of cultured cells by ELISA assay. It was found that only the MMP3 concentration was approximately 9.5 fold higher in the culture supernatant from BO2/MT3/LUC/PURO cells (mean 4.57±3.7, median 2.86), as compared to the BO2/LUC/PURO control cells (mean 0.48±0.5 ng/ml, median 0.22, p<0.0001; Mann–Whitney test) (**Fig 5F**). Three other metalloproteinases, MMP1, MMP2, and MMP9, were not detected in the culture media of either the analyzed cell types. When BO2/MT3/LUC/PURO cells and BO2/LUC/PURO control cells were also analyzed for the presence of these metalloproteinases by Western blot, the MMP1, MMP3 and MMP9 were detected in whole cell lysates. Interestingly, no differences in the amounts of these proteins were observed despite differences in expression of mRNA for MMP1 and MMP9 and mRNA and secreted protein in the case MMP3 (**S3 Fig**). Also decrease in TIMP1 mRNA (*p*<0.01) was observed in BO2/MT3/LUC/PURO in comparison with the BO2/LUC/PURO control cells (Mann–Whitney test) **(Fig 5E)**, but this was not confirmed when the TIMP1 was determined in the culture supernatants.

**Fig 6:**
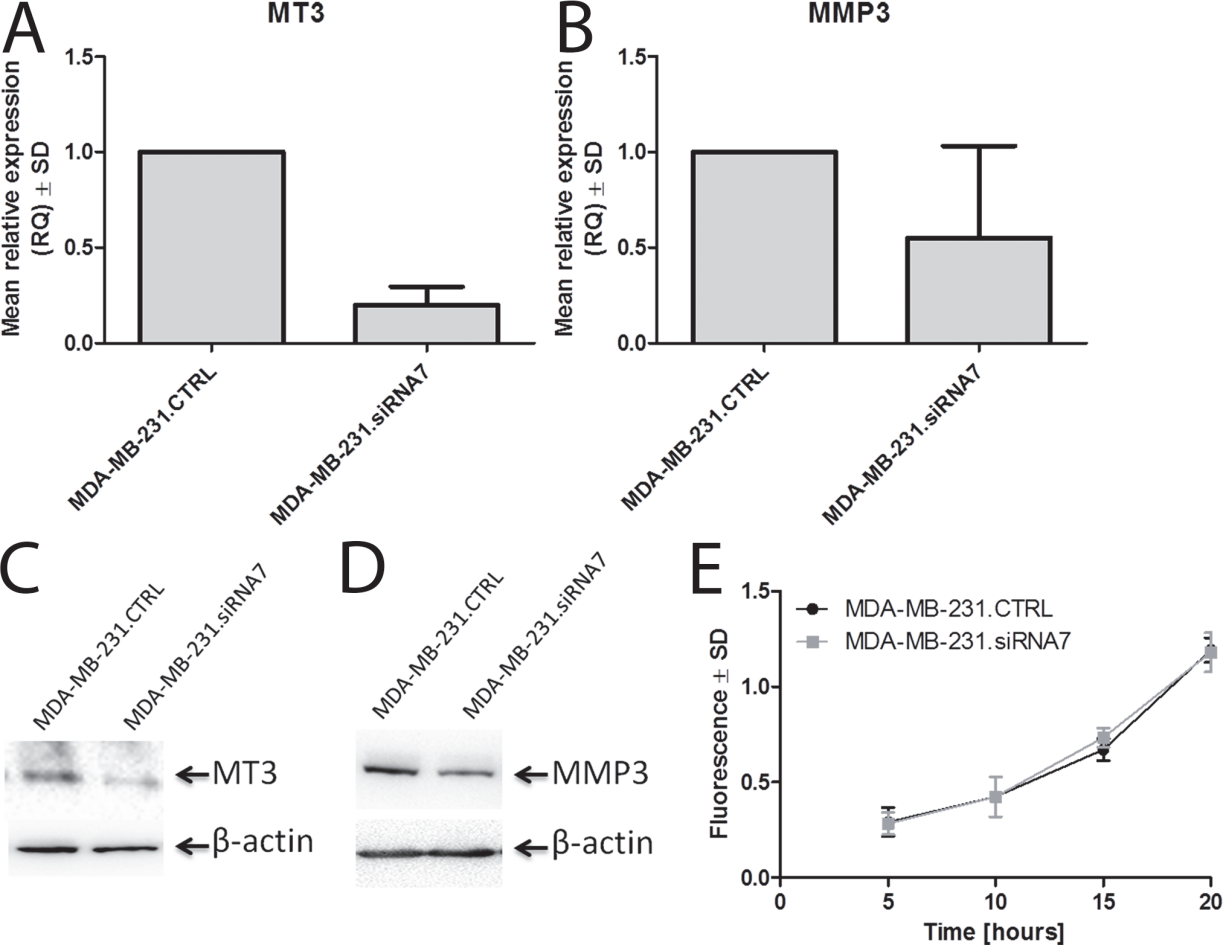
MT3 affects the expression of MMP3 in breast cancer MDA-MB-231 cells. Expression of MT3 mRNA **(A)** and MMP3 mRNA **(B)** in MDA-MB-231 cells treated with scrambled siRNA (MDA-MB-231.CTRL) and MDA-MB-231 cells treated with siRNA directed against MT3 mRNA (MDA-MB-231.siRNA7). Real-time PCR was used to analyze MT3 mRNA and MMP3 mRNA. Relative expression (RQ) of *MT3* and *MMP3* genes was normalized against expression of *ACTB* gene and MDA-MB-231.CTRL cells were assigned as a calibrator sample. Results are expressed as mean ± SD.

To further assess the possible relationship between MT3 overexpression and down-regulation of MMP3, we created a specific loss-of-function phenotype using siRNAs in order to inhibit the expression of MT3 in breast cancer MDA-MB-231 cells. Such cells, named MDA.MT3.siRNA7, were characterized by highly decreased levels of MT3 mRNA and highly decreased binding of anti-MT3 antibodies to cell lysates in comparison to control cells transfected with scrambled siRNA and named MDA.MT3.CTRL (**Fig 6A and 6B**). Importantly, when MDA-MB-231 cells were analyzed by real-time PCR and Western blot to assess, respectively, the level of MMP3 mRNA and protein expression, the down-regulation of MMP3 was observed in MDA.MT3.siRNA7 cells (**Fig 6C and 6D)**. However, when siRNA treated MDA-MB-231 cells with decreased expression of MT3 and down-regulation of MMP3, representing loss-of-function phenotype, and control MDA-MB-231 cells were subjected to *in vitro* matrigel invasion assay, no differences in invasive properties were found between these cells (**Fig 6E**).

Western blot analysis using anti-MT3 rabbit polyclonal antibodies **(C)** and anti-MMP3 monoclonal murine antibody **(D)** on whole cell lysates of MDA-MB-231 cells treated with scrambled siRNA (MDA-MB-231.CTRL) and MDA-MB-231 cells treated with siRNA directed against MT3 mRNA (MDA-MB-231.siRNA7). Cell lysates equivalent to 30 μg of protein were separated by SDS-PAGE under reducing conditions on a 12% gel and electrophoretically transferred onto a nitrocellulose membrane. β-Actin served as an internal control**. (E)** Invasiveness of MDA-MB-231 cells treated with scrambled siRNA (MDA-MB-231.CTRL) and MDA-MB-231 cells treated with siRNA directed against MT3 mRNA (MDA-MB-231.siRNA7). Data are presented as mean ± SD. Bonferroni multiple comparison test.

### MMP3 is responsible for the increased invasiveness of MT3-overexpressing breast cancer cells

**Fig 7:**
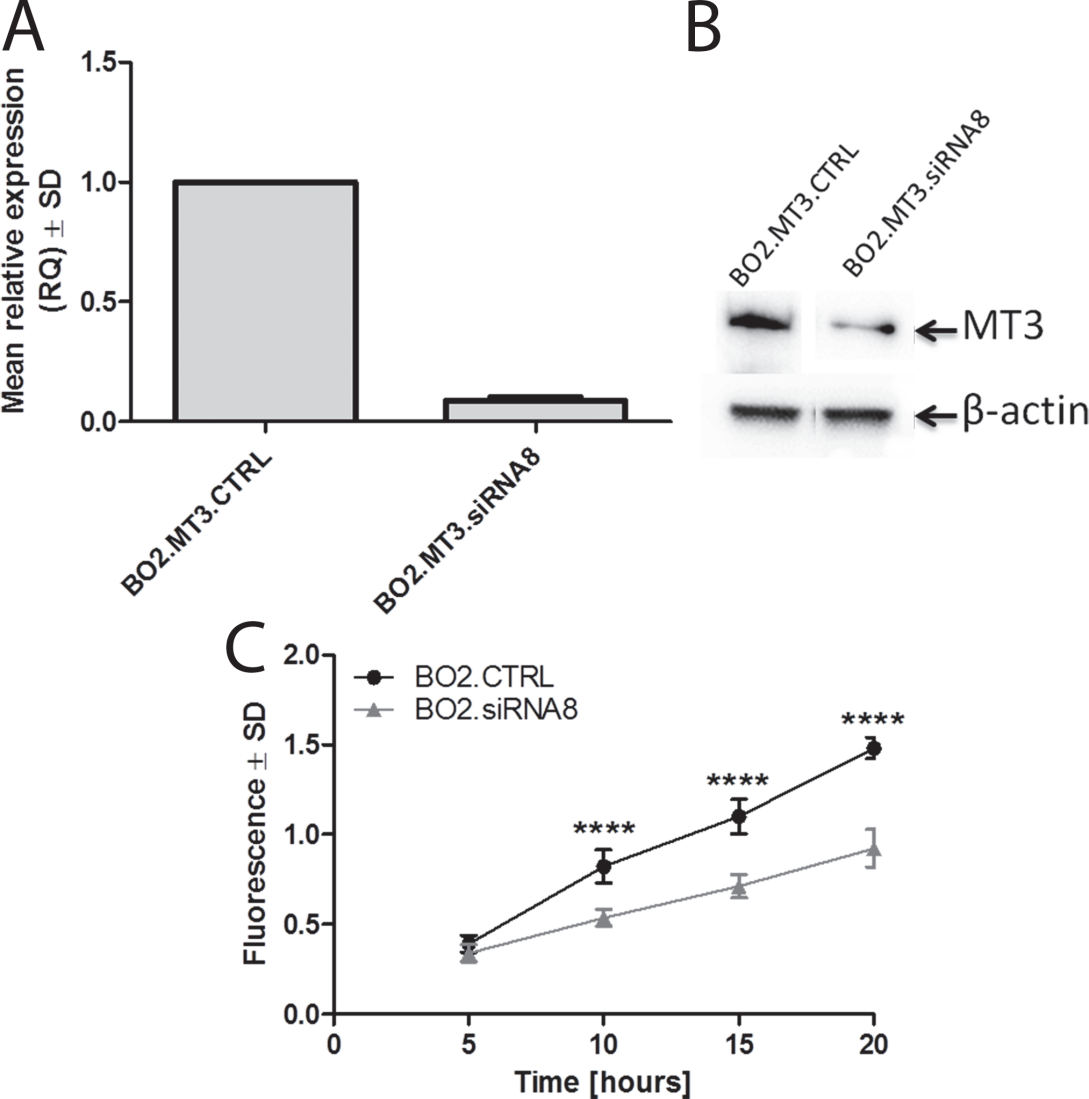
MMP3 is responsible for the increased invasiveness of MT3-overexpressing breast cancer cells. **(A)** Expression of MMP3 mRNA in control MDA-MB-231/BO2 cells transfected with scrambled siRNA (BO2.MT3.CTRL) and MDA-MB-231/BO2 cells treated with siRNA directed against MMP3 mRNA (BO2.MT3.siRNA8). Real-time PCR was used to analyze MT3 mRNA. Relative expression (RQ) of *MMP3* gene was normalized against expression of *ACTB* gene and BO2.MT3.CTRL cells was assigned as a calibrator sample. Results are expressed as mean ± SD. **(B)** Western blot analysis using anti-MMP3 monoclonal murine antibody on whole cell lysates of control MDA-MB-231/BO2 cells transfected with scrambled siRNA (BO2.MT3.CTRL) and MDA-MB-231 cells treated with siRNA directed against MMP3 mRNA (BO2.MT3.siRNA8). Cell lysates equivalent to 30 μg of protein were separated by SDS-PAGE under reducing conditions on a 12% gel and electrophoretically transferred onto a nitrocellulose membrane. β-Actin served as an internal control**. (C)** Invasiveness of control MDA-MB-231/BO2 cells transfected with scrambled siRNA (BO2.MT3.CTRL) and MDA-MB-231 cells treated with siRNA directed against MMP3 mRNA (BO2.MT3.siRNA8). Data are presented as mean ± SD. ****p<0.0001, Bonferroni multiple comparison test.

To further prove that MMP3 is directly involved in increased invasion of BO2/MT3/LUC/PURO cells, siRNA based RNA interference study was performed to inhibit the expression of *MMP3* gene. MT3-overexpressing MDA-MB-231/BO2 cells were incubated with specific siRNA and, after 48 h, analyzed by qPCR and Western blot for the presence of MMP3 mRNA and protein, respectively. It was found that one, out of 4 used siRNAs, silenced efficiently the expression of *MMP3* gene **(Fig 7A and 7B**). Such MT3-overexpressing MDA-MB-231/BO2 cells with highly decreased expression of MT3, named BO2.MT3.siRNA8, and the control BO2.MT3.CTRL cells with high expression of MMP3, transfected with scrambled siRNA cells, were subjected to *in vitro* matrigel invasion assay. A statistically significant decrease in invasiveness was found in the case of breast cancer cells with MMP3 expression silenced, in comparison to the control cells, at the 10^th^, 15^th^, and 20^th^ hours of the experiment (p<0.0001, p<0.0001 and p<0.0001, respectively; Bonferroni multiple comparison test) (**Fig 7C**).

### MT3 expression in TNBC samples and its correlation with patients’ clinical and pathological data

**Fig 8:**
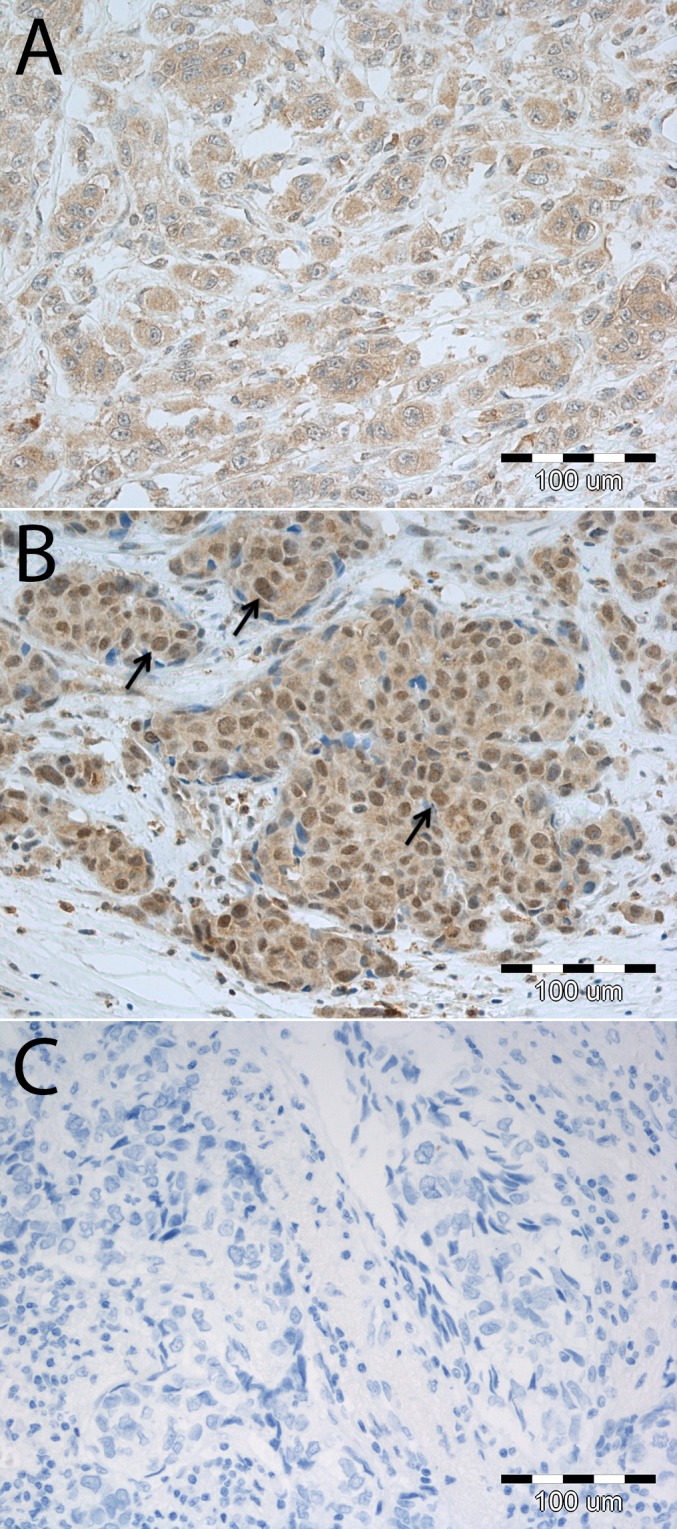
MT3 expression in triple-negative breast cancers (TNBC). **(A)** Cytoplasmic and (**B**) cytoplasmic-nuclear MT3 immunoreactivity in cancer cells. Arrows indicate nuclear MT3 localization. **(C)** No color reaction could be observed in case of negative controls performed by omitting the primary antibodies. Magnification ×200.

**Fig 9:**
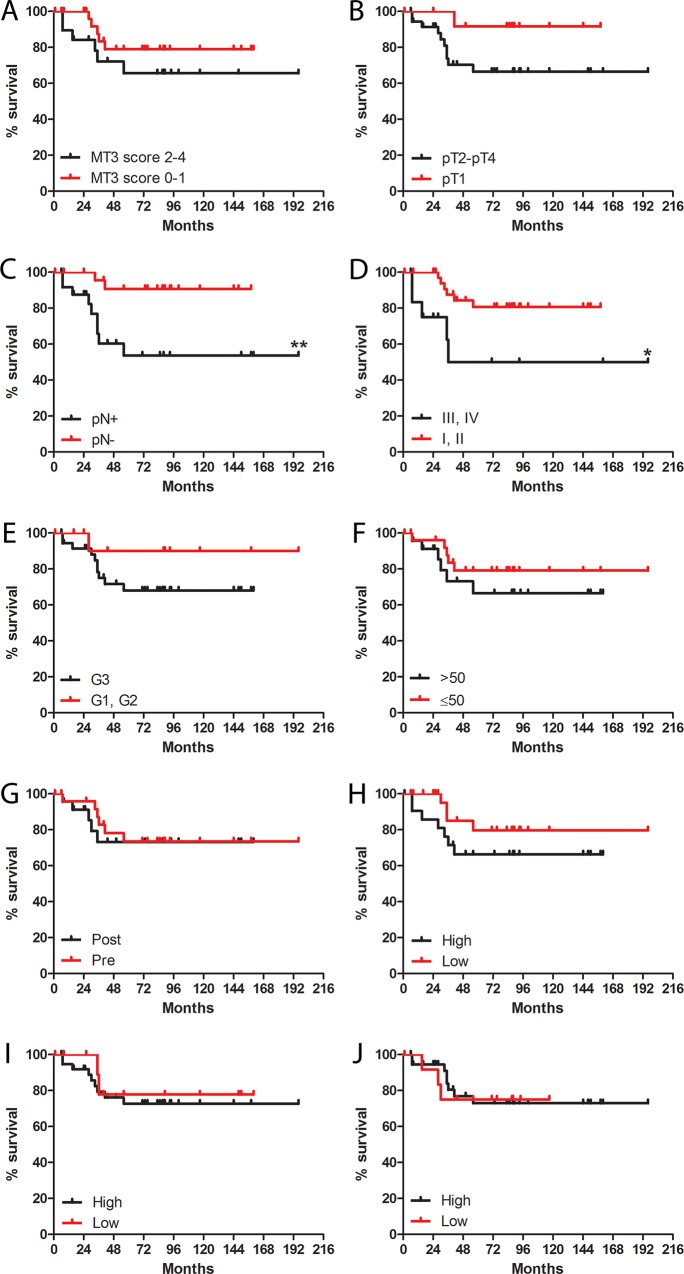
Univariate analysis of triple-negative breast cancer (TNBC) patients. Kaplan-Meier disease specific survival curves in regard to MT3 nuclear expression, primary tumor size (**B**), lymph node status (**C**), advancement stage (**D**), malignancy grade (**E**), patients age (**F**), menopausal status (**G**), extent of necrosis (**H**), fibrosis (**I**) and lymphocytic infiltration (**J**). Mantel-Cox test, *p<0.05, **p<0.01.

In the TNBC tumor sections, MT3 immunoreactivity was observed in the nuclei as well as in the cytoplasm of the cancer cells. Nuclear positivity was noted in 42 (82.4%) cases, of which 23 tumors were scored as “1”, ten as “2”, five as “3” and four as “4” (**Fig 8**). No association was found between MT3 expression intensity and extent of necrosis, fibrosis and tumor infiltrating lymphocytes. A higher nuclear MT3 expression was noted in pT2-pT4 (mean 1.62±1.21, median 1.0) as compared to pT1 (mean 1.00±0.78, median 1.0) tumors. To note, TNBC cases of pT3-pT4 were characterized by the highest nuclear MT3 expression (mean 2.76±1.77, median 3.0). In addition, tumors in advanced disease stages (III, IV; mean 1.92 ± 1.55, median 2.0), in comparison to less advanced tumors (I, II; mean 1.29±0.93, median 1.0), were characterized by higher MT3 nuclear expression. However, the differences did not reach the threshold of statistical significance (p>0.05). No differences in MT3 expression in nuclei of cancer cells could be noted in regard to lymph node status, patients age and menopausal status. Univariate survival analysis revealed that nuclear MT3 immunoreactivity of analyzed TNBC was not a prognostic factor, however cases scored “2” and higher were characterized by trend towards a shorter disease-specific survival (**Fig 9**). The presence of lymph-node metastasis (p<0.01) and advanced disease stage (III, IV; p<0.05) were associated with shorter disease-specific survival of patients (Table 2).

**Table 2:** Univariate survival analysis in 51 triple negative breast cancer (TNBC) patients. Significant p-values are given in bold print.

Clinical or pathological parameter	Disease-specific survival		
	HR	95% CI	p-value
MT3 (0–1 pts. *vs*. 2–4 pts.)	2.007	0.5913–6.810	0.2639
Age (≤50 *vs*. >50)	1.805	0.5360–6.079	0.3404
Menopause (yes *vs*. no)	1.189	0.3540–3.991	0.7797
Tumor size (pT1 *vs*. pT2-pT4)	2.966	0.8329–10.56	0.0934
Lymph nodes (pN- *vs*. pN+)	5.489	1.631–9.513	**0.0060**
Stage (I, II *vs*. III-IV)	6.526	1.387–30.72	**0.0176**
Malignancy grade (G1, G2 *vs*. G3)	2.486	0.6498–9.513	0.4989
Necrosis (absent-low *vs*. moderate-intense)	2.124	0.6436–7.007	0.2163
Fibrosis (absent-low *vs*. moderate-intense)	1.340	0.3288–5.458	0.6833
Tumor infiltrating lymphocytes (absent-low *vs*. moderate-intense)	0.9686	0.2528–3.711	0.9629

Abbreviations: HR, hazard ratio; CI, confidence interval; MT3, metallothionein-3

## Discussion

Recent studies have shown that MT3 is overexpressed in breast, urothelial, prostate, and non-small cell lung cancers; however, downregulation of MT3 has been observed in gastric and esophageal cancers [13,15–19,29]. In breast cancer, MT3 was found in the cytoplasm of cancer cells, with no expression in normal breast epithelial cells, and intensive MT3 staining was associated with patient poor outcome [15,29]. In contrast to the MT1/2 isoforms [30,31], the role of MT3 in cancer progression is poorly understood, including breast cancer. Therefore, the present study was undertaken to evaluate the role of MT3 in breast cancer cell proliferation, tumorigenesis, migration, and invasiveness. The analysis of cultured non-cancerous (hTERT-HME1) and breast cancer cell lines (MCF-7, SK-BR-3, BT-474, MDA-MB-231 and MDA-MB-231/BO2) revealed the presence of high levels of MT3 in the cytoplasmic fractions of hTERT-HME1 and MDA-MB-231 cells, and lower amounts in the cytoplasmic fractions of SK-BR-3 and BT-474 cells. Only residual amounts of MT3 were found in their nuclear fractions. Therefore, our study, which shown that MT3 is mainly localized in the cell cytoplasm, agrees with the results obtained by others for cancerous tissues [14,15,29]. There was no expression of MT3 in MDA-MB-231/BO2 cells on the level of protein and mRNA. Our finding contrasts with the results obtained by Gurel *et al*., who did not observe MT3 expression in any of the four breast cancer cell lines analyzed by them (MCF-7, MDA-MB-231, T47D, Hs578T) [22]. These contradictory results obtained for MCF-7, T47D and Hs578T cells with low expression of MT3 might be explained by the use of more sensitive real-time PCR in this work in comparison to conventional RT-PCR. However, we do not have explanation for the discrepancies observed in the case of MDA-MB-231 cells with high expression of MT3. In addition, we have noted MT3 expression in the immortalized breast epithelial hTERT-HME1 cells, which disagrees with the conclusions of Sens *et al*. and Somji *et al*., who did not find MT3 in normal breast epithelium [15,29]. We speculate that immortalization of these cells could lead to the observed MT3 upregulation.

The further investigation proceeded by constructing a specific “gain-of-function” phenotype represented by MDA-MB-231/BO2 cells transduced with MT3 cDNA. These cells were chosen, as they do not express MT3, unlike all the other analyzed breast cancer cell lines. Therefore, it was the only cell line which was appropriate to develop cellular model with ectopic expression of MT3. The MDA-MB-231/BO2 cells were originally selected to grow in bone after cardiac transplantation [25]. However, our assumption was that, if cells with ectopic expression of MT3 have different properties in comparison to parental cells (independently of their metastatic properties), than these differences can be linked to the presence of MT3.

MT3 was known originally by the name Growth Inhibitory Factor (GIF), as it possesses growth inhibitory activity [32]. In the case of breast cancer, overexpression of MT3 inhibits the growth of MCF-7 and Hs587T cells, but not T47D, MDA-MB-231, or ZR-75 cells [22]. Contradictory results were also obtained for prostate cancer cells. In the study of Dutta *et al*., overexpression of MT3 led to growth inhibition of PC-3 prostate cancer cells [23], although, in the recent study of Juang *et al*., MT3 overexpression in the same cell line resulted in augmented cell proliferation and tumorigenesis [24]. They attempted to explain this discrepancy by the predominance of the nuclear expression of MT3 in their PC-3 cells [24]; however, there are no indications that the localization of MT3 was different in the earlier studies. Interestingly, overexpression of MT3 in MDA-MB-231/BO2 breast cancer cells resulted in the appearance of MT3 not only in cytoplasm, but also in cell nuclei, which is in agreement with the above results [24]. However in our study, the overexpression of MT3 did not affect either the growth rate of MDA-MB-231/BO2 cells *in vitro* nor their tumorigenic properties *in vivo*, as the volumes of the tumors formed by the control BO2/LUC/PURO and BO2/MT3/LUC/PURO cells were similar. In accordance with the above findings, immunohistochemical staining of tumor specimens revealed that high expression of MT3 is neither associated with higher proliferative index nor with low numbers of apoptotic cells. It is also noteworthy that Gurel *et al*. did not observe any significant impact of MT3 overexpression on the proliferation rate of MDA-MB-231 cells [22], which are parental to MDA-MB-231/BO2 cells [25]. Taken together, it seems possible that effect of MT3 overexpression on the growth rate of breast cancer cells is dependent on cell type; however, the exact molecular mechanisms underlying this effect are currently unknown.

So far, the overexpression of MT3 in breast cancer cells has not been linked to their invasive properties. However, enhanced cell invasiveness has been observed when prostate cancer PC-3 cells were forced to overexpress MT3 [24]. In addition, it has been recently shown that high expression of metallothionein-2A (MT2A) in MDA-MB-231 cells is associated with their increased invasiveness [28]. In line with these findings, when our MDA-MB-231/BO2 cells overexpressing MT3 were subjected to invasion assay, a large increase in their invasiveness was observed in comparison to the control MDA-MB-231/BO2 cells.

It has been shown that the change in the invasive potential of MDA-MB-231 cells with overexpression of MT2A was most probably associated with the increased expression of MMP9; several other lines of evidence also suggest that MT1/2 may regulate the expression of MMPs and TIMPs in various tissues [33–37]. For example, it has been demonstrated that MT1E expression correlates positively with the invasive and migratory abilities of glioma cells. However, MMP and TIMP expression levels have not been studied in order to elucidate the potential mechanisms of this finding [37]. An increase in MMP2 expression has been observed in peripheral lung epithelial cells as result of treatment with cadmium, a potent MT1/2 inducer. Indeed, the observed effects were accompanied by a simultaneous increase in MT1/2 expression [36]. Furthermore, in transgenic *MT/RET* mice characterized by the development of MT-overexpressing malignant melanomas, an increase in MMP2 and a decrease in TIMP2 expression has been noted [33]. For this reason, we analyzed the presence of several metalloproteinases and TIMP1 in the culture supernatants of BO2/LUC/PURO and BO2/MT3/LUC/PURO cells. It was found that the presence of MT3 in BO2/MT3/LUC/PURO cells significantly increased the mRNA expression levels of *MMP1*, *MMP2*, *MMP3*, and *MMP9*, with a concomitant decrease in *TIMP1* expression. However, these results were confirmed on the protein level only for MMP3, which was found highly elevated in the culture media of BO2/MT3/LUC/PURO cells expressing MT3. Interestingly, in contrast to culture media, there was no differences in amounts of MMP3 between lysates of BO2/LUC/PURO and BO2/MT3/LUC/PURO cells. To explain this discrepancy, we propose that both cell types produce some amounts of MMP3, which can be detected inside the cells, however, all additional amounts of this protein produced by BO2/MT3/LUC/PURO cells are immediately secreted. No differences in amounts of MMP1 and MMP9 can be explained in the same way, but it is possible that the amounts of metalloproteinases secreted by the cells are probably too small to be detected by ELISA assay.

Direct evidence that MMP3 is responsible for increased invasiveness came from the experiment with siRNA-mediated inhibition of this matrix metalloproteinase in MT3-overexpressing MDA-MB-231/BO2 cells. Using *in vitro* matrigel invasion assay, a statistically significant decrease in invasiveness was found in the case of breast cancer cells with MMP3 expression silenced, in comparison to the control cells. Direct interdependence between the increased expression of MMP3 and overexpression of MT3 was further confirmed by transfecting human breast cancer MDA-MB-231 cells with siRNA to silence the expression of MT3. In this case, the decreased expression of MT3 was associated with decreased expression of MMP3. However, we did not observe the decrease in invasiveness of siRNA-treated MDA-MB-231 cells in comparison to control MDA-MB-231 cells. It is most probably associated with higher level of MMP3 in cells treated with siRNA directed against MT3, than in cells incubated with siRNA to directly silence the expression of MMP3.

It may well be asked why the increased invasiveness *in vitro* of the MDA-MB-231/BO2 cells expressing MT3 did not affect the tumor invasiveness *in vivo*, following transplantation into nude mice, as we did not note any differences in the infiltration patterns between the analyzed cell lines. This discrepancy can be explained by the fact, that tumors generated in the experimental setting were surrounded by a fibrous capsule until the 10^th^ week of the experiment, and in contrast to human TNBC samples, were deprived of microenvironment and immune infiltrate. As the cancer invasion is heavily dependent on tissue microenvironment [38], it is probably the reason why we did not find differences between MT3-overexprssing MDA-MB-231/BO2 cells and control MDA-MB-231/BO2 cells in their invasive properties *in vivo*. Therefore, our data show also the limitations of nude mice model in studies on cancer invasion.

To link the observed increased invasiveness of the triple-negative MDA-MB-231/BO2 breast cancer cells overexpressing MT3 with the clinical situation, we analyzed MT3 expression in 51 cases of TNBC. It was found that the increased nuclear MT3 immunoreactivity of breast cancer cells correlated with the more aggressive phenotype, as the expression of MT3 reached its highest values in the most locally advanced tumors (pT3-pT4), and was more pronounced in cases at advanced disease stages. Furthermore, patients with high nuclear MT3 expression tended to have a shorter disease-specific survival, as compared to the cases showing only cytoplasmic MT3 immunoreactivity. Although the study group consisted of only 51 TNBC cases, the well-established clinical and pathological parameters, such as presence of lymph node metastasis and advanced disease stage, were also associated with poor patient outcome.

In our recent study, nuclear MT3 expression was also observed in pneumocytes and cancer cells of non-small cell lung cancers. Unlike the results of the current study, Werynska *et al*. observed a decrease in nuclear MT3 immunoreactivity in cancer cells with increasing malignancy grade of the tumors [16]. Interestingly, other studies that have analyzed the immunoreactivity of MT3 in tumor sections have only reported its cytoplasmatic localization [14,15,29]. Using immunohistochemical methods, increased cytoplasmatic MT3 expression was identified in human urinary bladder and breast cancer [14,29]. Due to the lack of studies analyzing MT3 expression in human cancer tissues, further research on human tumors seems to be justified, with the aim of elucidating the significance of varying MT3 cellular localizations on tumor progression.

In summary, we have shown that overexpression of MT3 in breast cancer cells increases their invasiveness, most probably *via* upregulation of MMP3 activity. In addition, our data point to the negative prognostic impact of nuclear MT3 expression in cancer cells of triple-negative breast cancer.

## Supporting Information

S1 Fig Western blot analysis of nuclear and cytoplasmic fractions of HME1-hTERT and breast cancer cells lysates using rabbit polyclonal antibodies directed against histone H3 (nuclear protein) and calpain (cytoplasmic protein). Cell lysates equivalent to 30 μg of protein were separated by SDS-PAGE under reducing conditions on a 12% gel and electrophoretically transferred onto a nitrocellulose membrane.
(TIF)

S2 Fig Immunohistochemical analysis of tumor xenografts of control BO2/LUC/PURO cells and BO2/MT3/LUC/PURO cells overexpressing MT3. **(A)** Immunohistochemical staining with monoclonal antibody against Ki-67 antigen of tumor sections after subcutaneous implantation of control BO2/LUC/PURO cells (1) and MT3 BO2/MT3/LUC/PURO cells overexpressing MT3 (2) into nu/nu mice. The numbers of Ki67-positive cells were compared with the Mann–Whitney U-test. **(B)** TUNEL technique following subcutaneous implantation of BO2/LUC/PURO cells (1) and MT3 BO2/MT3/LUC/PURO cells (2) into nu/nu mice. The numbers of apoptotic cells were compared as above. Magnification ×400.
(TIF)

S3 Fig Western blot analysis of MMP1, MMP3, and MMP9 in whole cell lysates of control BO2/LUC/PURO cells and BO2/MT3/LUC/PURO cells overexpressing MT3. Cell lysates equivalent to 30 μg of protein were separated by SDS-PAGE under reducing conditions on a 12% gel and electrophoretically transferred onto a nitrocellulose membrane. The anti-MMP9 antibody (Dako) recognized several bands corresponding to MMP9 dimer (dMMP9), TIMP-MMP9 complex, pro-MMP9 (pMMP9) and active form of MMP9 (aMMP9). β-Actin served as an internal control.
(TIF)
