# Supplementary figures and images for: Metallothionein-3 Increases Triple-Negative Breast Cancer Cell Invasiveness via Induction of Metalloproteinase Expression
Source: PLoS One. 2015 May 1;10(5):e0124865. doi: 10.1371/journal.pone.0124865 (PMC4416915; doi:10.1371/journal.pone.0124865)

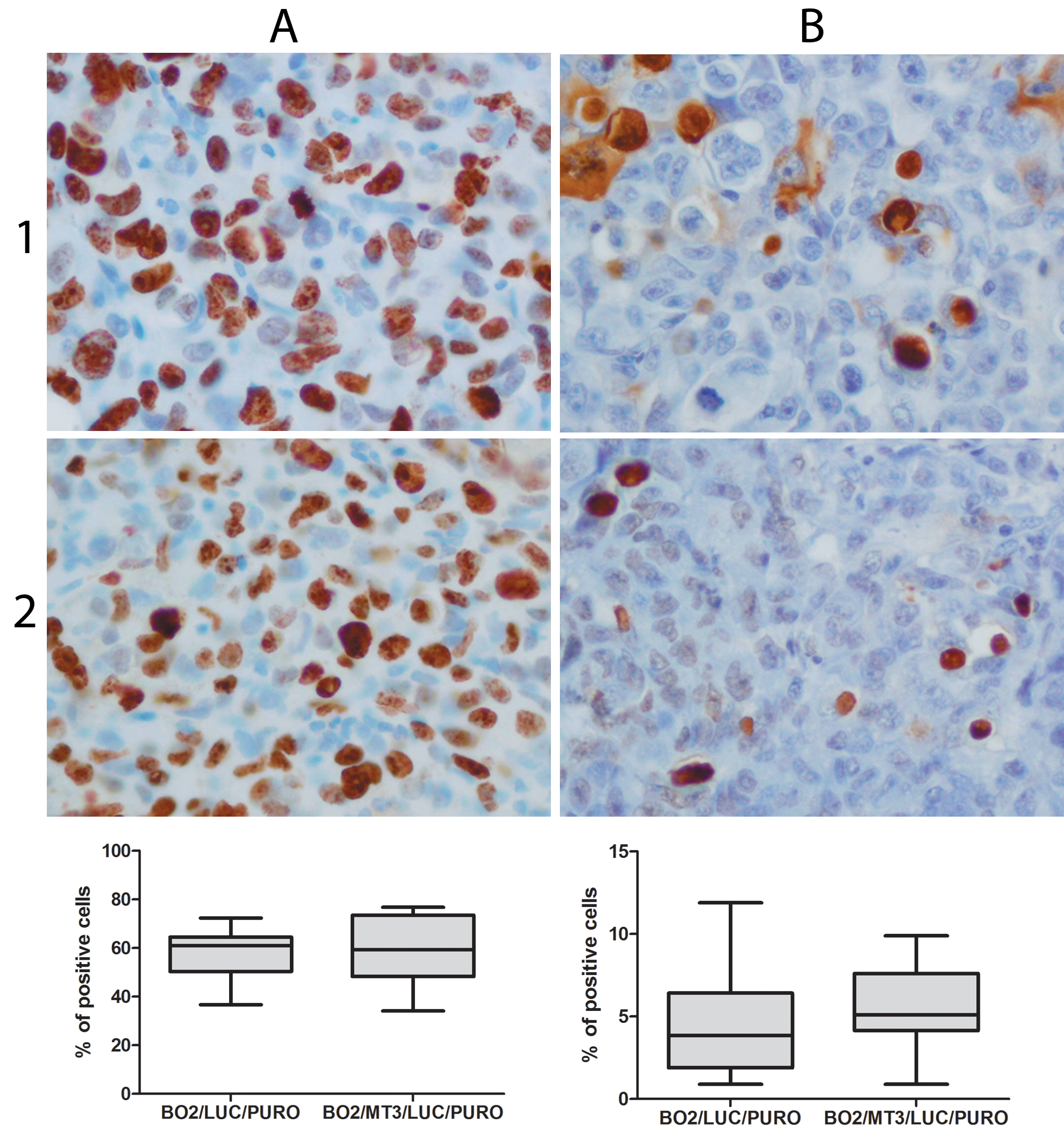

Supplement: S1 Fig — Cell lysates equivalent to 30 μg of protein were separated by SDS-PAGE under reducing conditions on a 12% gel and electrophoretically transferred onto a nitrocellulose membrane. (TIF) [file pone.0124865.s001.tif]

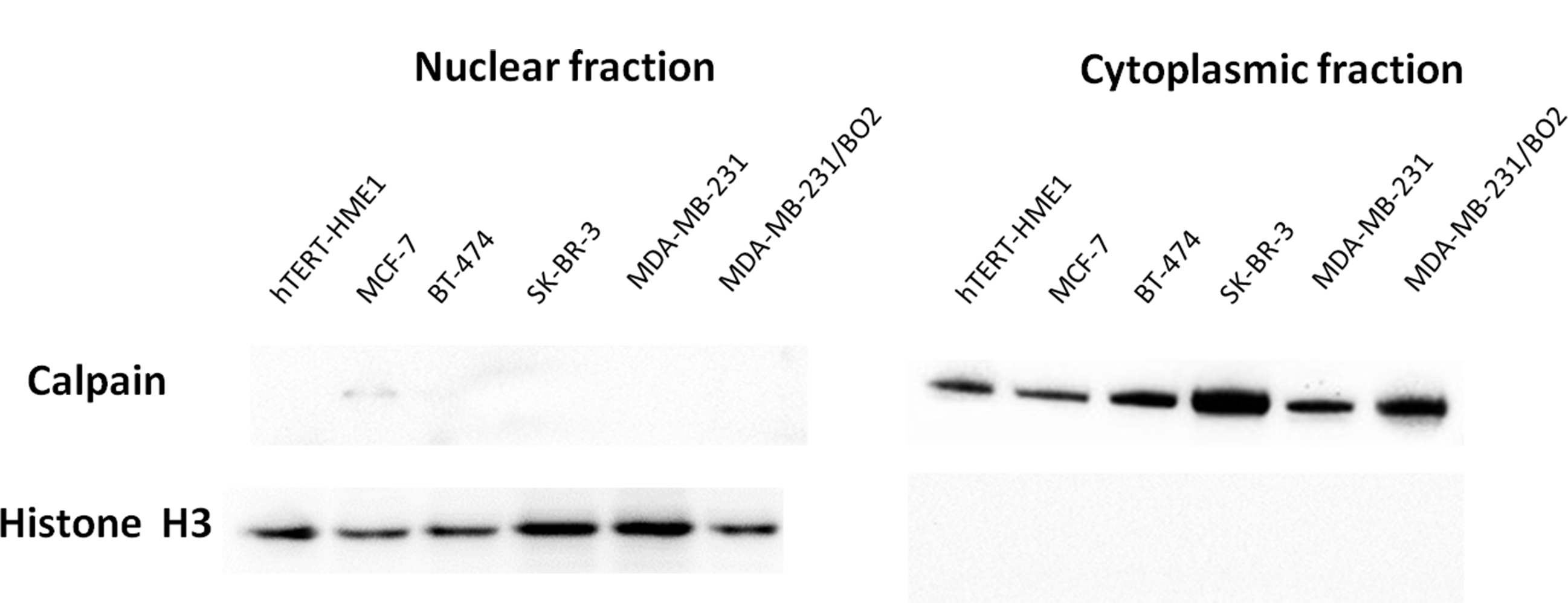

Supplement: S2 Fig — (A) Immunohistochemical staining with monoclonal antibody against Ki-67 antigen of tumor sections after subcutaneous implantation of control BO2/LUC/PURO cells (1) and MT3 BO2/MT3/LUC/PURO cells overexpressing MT3 (2) into nu/nu mice. The numbers of Ki67-positive cells were compared with the Mann–Whitney U-test. (B) TUNEL technique following subcutaneous implantation of BO2/LUC/PURO cells (1) and MT3 BO2/MT3/LUC/PURO cells (2) into nu/nu mice. The numbers of apoptotic cells were compared as above. Magnification ×400. (TIF) [file pone.0124865.s002.tif]

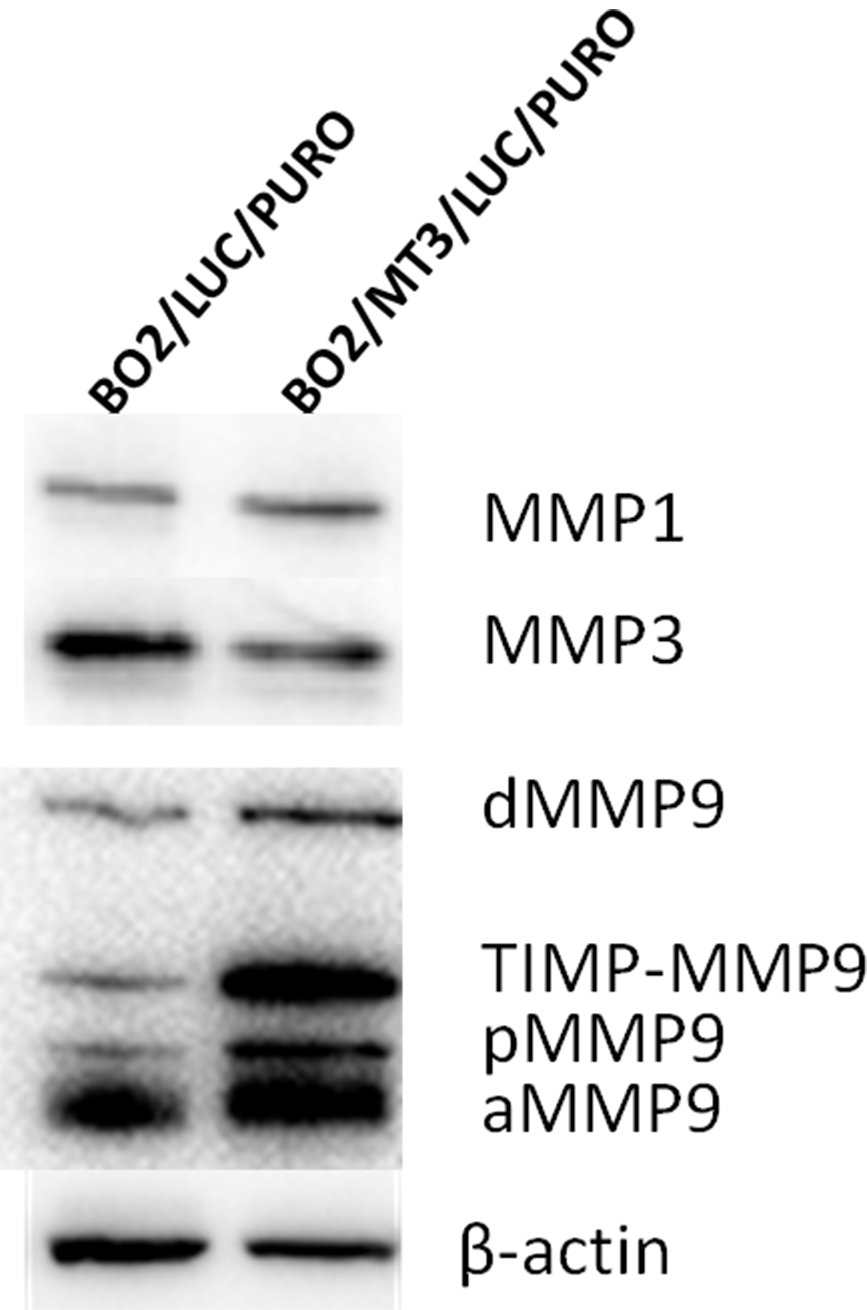

Supplement: S3 Fig — Cell lysates equivalent to 30 μg of protein were separated by SDS-PAGE under reducing conditions on a 12% gel and electrophoretically transferred onto a nitrocellulose membrane. The anti-MMP9 antibody (Dako) recognized several bands corresponding to MMP9 dimer (dMMP9), TIMP-MMP9 complex, pro-MMP9 (pMMP9) and active form of MMP9 (aMMP9). β-Actin served as an internal control. (TIF) [file pone.0124865.s003.tif]
